# Targeting fibroblast growth factor (FGF)-inducible 14 (Fn14) for tumor therapy

**DOI:** 10.3389/fphar.2022.935086

**Published:** 2022-10-21

**Authors:** Olena Zaitseva, Annett Hoffmann, Christoph Otto, Harald Wajant

**Affiliations:** ^1^ Division of Molecular Internal Medicine, Department of Internal Medicine II, University Hospital Würzburg, Würzburg, Germany; ^2^ Department of General, Visceral, Transplantation,Vascular and Pediatric Surgery, University Hospital Würzburg, Würzburg, Germany

**Keywords:** agonistic antibodies, cell death, Fn14, NFκB, TNF, TWEAK

## Abstract

Fibroblast growth factor-inducible 14 (Fn14) is a member of the tumor necrosis factor (TNF) receptor superfamily (TNFRSF) and is activated by its ligand TNF-like weak inducer of apoptosis (TWEAK). The latter occurs as a homotrimeric molecule in a soluble and a membrane-bound form. Soluble TWEAK (sTWEAK) activates the weakly inflammatory alternative NF-κB pathway and sensitizes for TNF-induced cell death while membrane TWEAK (memTWEAK) triggers additionally robust activation of the classical NF-κB pathway and various MAP kinase cascades. Fn14 expression is limited in adult organisms but becomes strongly induced in non-hematopoietic cells by a variety of growth factors, cytokines and physical stressors (e.g., hypoxia, irradiation). Since all these Fn14-inducing factors are frequently also present in the tumor microenvironment, Fn14 is regularly found to be expressed by non-hematopoietic cells of the tumor microenvironment and most solid tumor cells. In general, there are three possibilities how the tumor-Fn14 linkage could be taken into consideration for tumor therapy. First, by exploitation of the cancer associated expression of Fn14 to direct cytotoxic activities (antibody-dependent cell-mediated cytotoxicity (ADCC), cytotoxic payloads, CAR T-cells) to the tumor, second by blockade of potential protumoral activities of the TWEAK/Fn14 system, and third, by stimulation of Fn14 which not only triggers proinflammtory activities but also sensitizes cells for apoptotic and necroptotic cell death. Based on a brief description of the biology of the TWEAK/Fn14 system and Fn14 signaling, we discuss the features of the most relevant Fn14-targeting biologicals and review the preclinical data obtained with these reagents. In particular, we address problems and limitations which became evident in the preclinical studies with Fn14-targeting biologicals and debate possibilities how they could be overcome.

## 1 Introduction

Fibroblast growth factor (FGF)-inducible 14 (Fn14) is an unusual small member of the tumor necrosis factor (TNF) receptor superfamily (TNFRSF) of 14 kDa (Wiley et al., 2001). TNFRSF receptors (TNFRs) are characterized by a cysteine rich domain (CRD) which is found in one to six copies in their N-terminal extracellular parts. Structurally three TNFR subgroups can be distinguished: 1) death receptors (DRs), which harbor a death domain (DD) in their cytoplasmic part mediating the assembly of receptor-associated or cytoplasmic caspase activating protein complexes (Dickens et al., 2012) 2) TRAF-interacting TNFRs, which have no DD but instead contain one or more binding motifs for adapter proteins of the TNF receptor associated factor (TRAF) family in their cytoplasmic domain and 3) decoy TNFRs, which lack an intracellular domain. Fn14 belongs to the second subgroup of TNFRs and binds TRAF1, TRAF2, TRAF3, and TRAF5 (Wiley et al., 2001).

Fn14 is dynamically and highly expressed during development. In contrast, in the healthy adult organism Fn14 expression is low and mainly limited to mesenchymal and epithelial progenitor cells. Importantly, however, rapid and strong upregulation of Fn14 expression takes place in practically any non-hematological cell type upon tissue injury (Winkles, 2008; Burkly et al., 2011). The injury-associated pattern of Fn14 expression is in good accordance with the fact that growth factors (e.g., EGF, FGF), various cytokines (e.g., IFNγ, TNF, TGFβ) but also microbial (e.g., LPS) and physical stressors (e.g., hypoxia, irradiation) are potent Fn14 inducers. In accordance with the expression pattern of Fn14, manifold and complex functions of Fn14 have been described in tissue repair and regeneration. For example, it has been found that regenerative responses after the injury of muscles, pancreas and the liver are delayed in Fn14 knockout mice (Girgenrath et al., 2006; Mittal et al., 2010a; Mittal et al., 2010b). Furthermore, excessive or chronic engagement of Fn14 may also promote repair-related adverse effects, such as fibrosis and chronic inflammation (Mittal et al., 2010a; Mittal et al., 2010b; Kuramitsu et al., 2013).

The tissue damage that is unavoidably associated with tumor growth typically comes along with sterile inflammation and triggering of repair processes. As a consequence, the tumor microenvironment contains a variety of the aforementioned Fn14-inducing factors and expression of Fn14 has been accordingly described for a huge variety of tumors of non-hematopoietic origin (Table 1).

**TABLE 1:** Cancer-associated expression of Fn14.

Tumor entity	Remark	References
Breast cancer	Fn14 expression correlated with poor prognosis	Willis et al. (2008)
	Association between Fn14 and HER2 expression	
	Fn14 expression in 148 of 171 cases (86.5%)	Wang et al. (2013)
Luminal breast carcinoma	Fn14 expression prognostic for brain metastasis	Martinez-Aranda et al. (2015), Martinez-Aranda et al. (2017)
Colorectal cancer	Fn14 expression in 75% (128/171) of primary colorectal tumors, in 76% (62/82) of colorectal tumors with liver metastases but only in 7% (2/29) of normal colorectal tissues	Culp et al. (2010)
Endometrial cancer	Fn14 upregulation	Wang et al. (2010)
Esophageal adenocarcinoma	Fn14 upregulation	Watts et al. (2007)
Esophageal cancer	Fn14 expression in 10 of 43 cases (23.2%)	Yoriki et al. (2011)
Gastric cancer	Fn14 expression inversely correlated with survival	Kwon et al. (2012)
Glioblastoma multiforme (GBM)	Fn14 expression increases with tumor grade	Tran et al. (2003)
Recurrent GBM, Gliosarcoma	Fn14 in recurrent GBM higher than in matched primary	Hersh et al. (2018a)
Glioma	Fn14 reduced disease-free survival	Tan et al. (2018), Connolly et al. (2021), Tao et al. (2022)
	Fn14 expression correlates with high plasminogen activator inhibitor-1 expression	
Neuroblastoma	Fn14 expression higher in primary high stage tumors compared to low stage tumors	Pettersen et al. (2013)
Head and neck squamous cell carcinoma	Fn14 expression associated with poorprognosis	Aye et al. (2021)
Hepatocellular cancer	Fn14 expression in 142 of 260 cases (54.6%)	Li et al. (2013)
Non-small cell lung cancer	48.6% of adenocarcinoma Fn14^+^	Whitsett et al. (2012)
	31.5% of squamous cell carcinoma Fn14^+^	
Non-small cell lung cancer	Fn14 expression associated with higher mortality	Wang et al. (2021)
Small cell lung cancer	Fn14 expression in 26 of 51 cases (50.98%)	Li et al. (2014)
	Fn14 expression associated with poor pathologic stage	
Melanoma	Fn14 expression in 173 of 190 cases (92%) of primary melanoma and in 86 of 150 cases (58%) of melanoma metastases	Zhou et al. (2013a)
Oral squamous cell carcinoma	90% positive	Acharya et al. (2019)
Ovarian cancer	Fn14 expression in 73% (48/66) of ovarian cancer but 0% (0/2) in normal ovarian tissue	Culp et al. (2010)
Ovarian cancer	Fn14 expression in 35 of 41 cases (85.37%)	Gu et al. (2013)
Pancreatic cancer	Fn14 expression in 35 of 51 cases (68.6 5) pancreatic cancer.	Yoriki et al. (2011)
Pancreatic cancer	Fn14 expression in 79% (37/47) of pancreatic tumors but only in 17% (1/6) of normal pancreatic tissues	Culp et al. (2010)
Prostate cancer	10% of normal prostate epithelium Fn14 + but >75% of metastatic samples Fn14^+^	Yin et al. (2014)
	Fn14 expression inversely correlated with androgen receptor expression	
Prostate cancer	High expression of Fn14 associated with higher prostate-specific antigen recurrence rate after radical prostatectomy	Huang et al. (2011)
Thyroid cancer	Fn14 expression associated cancer risk	Lian et al. (2020)

## 2 Fn14 signal transduction

Fn14 signaling is activated by two variants of tumor necrosis factor (TNF)-like weak inducer of apoptosis (TWEAK), membrane-bound TWEAK (memTWEAK) and soluble TWEAK (sTWEAK). TWEAK is a typical ligand of the TNF superfamily (TNFSF). Accordingly, it is a type II homotrimeric transmembrane protein (memTWEAK) from which a soluble, likewise trimeric, molecule (sTWEAK) can be released by furin protease-mediated proteolytic processing (Bodmer et al., 2002). An early study claimed that death receptor (DR3) is also a TWEAK receptor (Marsters et al., 1998), but we and others could not confirm this interaction (Schneider et al., 1999; Kaptein et al., 2000). The scavenger receptor CD163 has also been identified as a TWEAK receptor (Bover et al., 2007), but we did not observe this interaction in highly sensitive cellular binding assays (Fick et al., 2012). TWEAK expression has been described in a variety of cell lines and cell types by immunohistochemistry and RT-PCR. Doubtless detection of memTWEAK, however, has only been reported for monocytes, dendritic cells, NK cells and T-cells and a very few tumor cell lines (Nakayama et al., 2000; Kaplan et al., 2002; Kawakita et al., 2004; Felli et al., 2005; Maecker et al., 2005). Thus, it appears that sTWEAK is more abundantly expressed, compared to memTWEAK.

TWEAK received its name due to the initial observation that it induces apoptosis in a very few cell lines (Chicheportiche et al., 1997). As mentioned before, TNF receptor 1 (TNFR1) and other apoptosis inducing receptors of the TNFRSF trigger cell death signaling by means of a characteristic protein-protein interaction domain present in their cytoplasmic part, the so called death domain (DD). Fn14 does not contain a DD (Chicheportiche et al., 1997). Accordingly, early on we could show in the Kym-1 cell line that Fn14 has no own authentic ability to directly instruct the assembly of a caspase-8 activating protein complex and instead triggers apoptosis indirectly by stimulating the production of TNF which subsequently kills the Kym-1 cells *via* TNFR1 (Schneider et al., 1999). A similar indirect mechanism was later on reported for some of the other few cell lines undergoing TWEAK-induced apoptosis (Vince et al., 2008). Stimulation of TNF production is not sufficient for TWEAK-induced apoptosis. The latter furthermore requires a second Fn14-mediated process, which sensitizes for TNFR1-induced activation of caspase-8 and apoptosis (Schneider et al., 1999; Vince et al., 2008; Wicovsky et al., 2009). This sensitizing mechanism is based on the reduction of the cytoplasmic TNFR1-available pool of TRAF2 and the TRAF2-associated molecules cIAP1 and cIAP2 by receptor recruitment. Originally this process has been identified as a mechanism by which TNFR2 enhances TNFR1-induced apoptosis (Duckett and Thompson, 1997; Weiss et al., 1997; Weiss et al., 1998). The concerted action of TRAF1, TRAF2 and the cIAPs attenuates the ability of TNFR1 to activate caspase-8 (Wang et al., 1998) Therefore, the sequestration of these antiapoptotic molecules by Fn14 enhances TNF-induced apoptosis. Efficient recruitment of TRAF2, and under some circumstances TRAF2 degradation, is the most receptor proximal event triggered by Fn14 activation and thus operates also in cells which do not produce TNF in response to Fn14 stimulation (Wicovsky et al., 2009). Consequently, TWEAK enhances the apoptotic effect of exogenous TNF also in cells in which TWEAK alone is unable to trigger cell death. Membrane TWEAK as well as soluble TWEAK trigger TRAF2 recruitment to Fn14, accordingly both TWEAK variants enhance TNF-induced apoptosis.

TNFR1, by help of the redundant involvement of the TNFR1-associated signaling molecules TRADD and RIPK1 (Fullsack et al., 2019), stimulates apoptotic cell death by triggering the formation of a cytosolic caspase-8 activating complex, called complex II (Micheau and Tschopp, 2003; Ting and Bertrand, 2016; Siegmund et al., 2017). Both, TRADD and RIPK1, can independently recruit TRAF2 and the cIAPs to the plasma membrane-associated TNFR1 signaling complex but also to the cytosolic caspase-8 activating complex. This way, the antiapoptotic TRAF2-cIAP1/2 complexes gain physically access to the caspase-8 activating protein machinery. Noteworthy, caspase-8 activation in context of apoptosis-induction by the TNFR1-related death receptors TRAILR1, TRAILR2 and CD95 occurs directly at the initially assembled plasma membrane associated receptor complexes. Since in these cases TRAF2 and cIAPs have less efficient access to caspase-8, apoptosis induction by these death receptors remains largely unaffected by TWEAK/Fn14-dependent TRAF2-cIAP1/2 sequestration. Intriguingly, we and others could show that TWEAK and Fn14 also enhance necroptosis triggered by various insults (Wicovsky et al., 2009; Karl et al., 2014; Anany et al., 2018; Martin-Sanchez et al., 2018). Necroptosis is a form of cell death that can be triggered in RIPK3-expressing cells especially when caspase-8 activation is suppressed. Necroptosis induction depends on the formation of a cytosolic RIPK1- and RIPK3-containing protein complex and deubiquitination of linearly- and K63-ubiquitinated RIPK1. Noteworthy, K63-ubiquitination of RIPK1 in context of TNFR1 signaling is performed by cIAP1 and cIAP2. Therefore, it is not surprising that TWEAK/Fn14-mediated sequestration of TRAF2-cIAP1/2 complexes enhances TNF-induced necroptosis. Other than in the case of caspase-8 activation, TNFR1, CD95 and the TRAIL death receptors do not differ in the use of the RIPK1-RIPK3 core complex for necroptosis triggering and this also applies to necroptosis induction by double-stranded RNA and TLR3 (Karl et al., 2014; Anany et al., 2018). Accordingly, TWEAK has been found to enhance necroptosis induction by all these triggers (Karl et al., 2014; Anany et al., 2018). The physiological and/or pathophysiological relevance of the apoptosis- and necroptosis-enhancing activity of the TWEAK-Fn14 system has been poorly investigated yet *in vivo*. There is, however, evidence that it contributes *in vivo* to TNF-driven cell death of intestinal epithelial cells and the delayed phase of cell death in acute kidney injury (Chopra et al., 2015; Grabinger et al., 2017; Martin-Sanchez et al., 2018). Moreover, TRAF2 and the cIAPs have recently been identified as major targets facilitating CD8^+^ T-cell elimination of tumor cells in a TNF-dependent manner *in vivo* (Vredevoogd et al., 2019). Therefore, Fn14-dependent TRAF2-cIAP1/2 sequestration could also be a process that could be exploited for tumor therapy (see also below).

TRAF2 along with cIAP1 and cIAP2 are not only of relevance in the regulation of DR-induced cell death signaling pathways but play also a complex role in the activation of signaling pathways culminating in nuclear translocation of transcription factors of the NF-κB family and transcription of NF-κB-regulated genes. The transcription factors of the NF-κB family are dimeric and are sequestered in the cytosol (Baeuerle and Baltimore, 1988a; Baeuerle and Baltimore, 1988b; Henkel et al., 1992; Rice et al., 1992; Huang et al., 1997; Sun, 2017; Zhang et al., 2017). Cytosolic sequestration of NF-κB dimers is largely based on masking of their nuclear localization sequence (NLS). The latter can be achieved either by intramolecular interaction with an ankyrin-repeat containing inhibitory domain which is present in two NF-κB proteins that are initially expressed as immature precursor molecules (p100, p105) or by intermolecular interaction with ankyrin-repeat containing IκB proteins (Baeuerle and Baltimore, 1988a; Baeuerle and Baltimore, 1988b; Henkel et al., 1992; Rice et al., 1992; Sun, 2017; Zhang et al., 2017) According to the signaling mechanisms that result in demasking of the NLS two fundamentally different NF-κB signaling pathways can be distinguished, the classical or canonical NF-κB pathway and the alternative or noncanonical NF-κB pathway (Sun, 2017; Zhang et al., 2017). Intriguingly, TRAF2, cIAP1 and cIAP2 play a crucial role in both signaling pathways but with a qualitatively opposing effect. In the classical NF-κB pathway, TRAF2 and the cIAPs are crucially involved in the receptor/signal-induced activation of the so called IKK complex which phosphorylates IκB proteins to trigger their degradation and p105 to accelerate its constitutive processing to mature p50 (Sun, 2017; Zhang et al., 2017). In contrast, TRAF2 and the cIAPs in concert with TRAF3 inhibit in unstimulated cells the alternative NF-κB pathway by K48-ubiquitination and proteasomal degradation of the NF-κB inducing kinase (NIK), a constitutively active kinase of the MAP3K family. NIK is able to stimulate the phosphorylation and activation of IKK1, which in turn triggers proteolytic processing of the inactive p100 NF-κB precursor protein to the NF-κB subunit p52 (Sun, 2017; Zhang et al., 2017).

**FIGURE 1:**
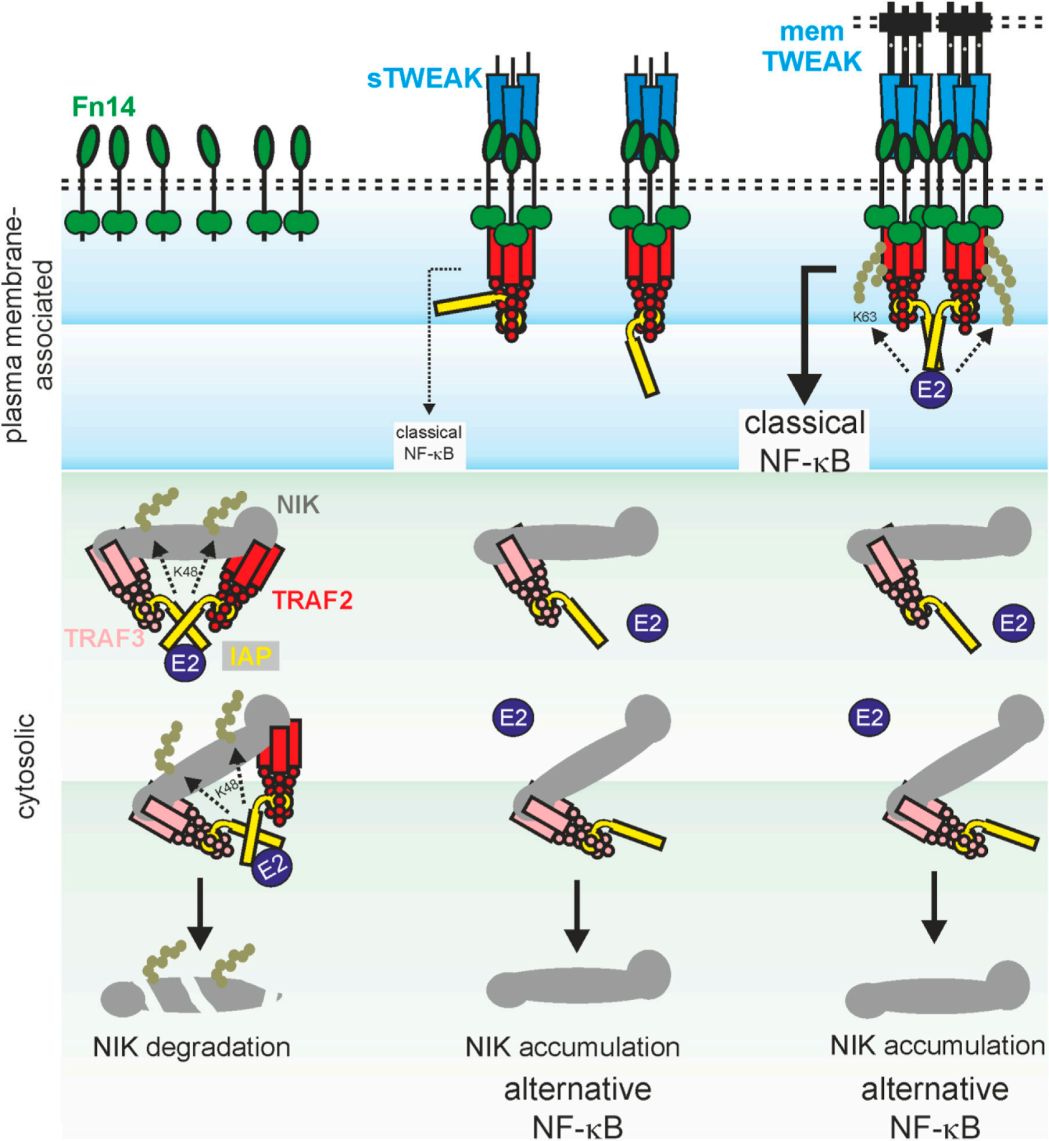
Model of TWEAK-induced NF-κB signaling. Dependent on the interplay of sTWEAK, memTWEAK, Fn14, TRAF-cIAP1/2 and TRAF3-cIAP1/2-NIK complexes, three different states of NFκB signaling can be distinguished: i) In the absence of TWEAK, TRAF3-cIAP1/2-NIK complexes interact with TRAF2-cIAP1/2 complexes in the cytoplasm. This results in cIAP1/2 transactivation and by help of E2 proteins to K48 ubiquitination of NIK by the cIAPs, proteasomal degradation of NIK and thus in constitutive inhibition of the alternative NF-κB pathway (left panel). ii) In the presence of sTWEAK, ligated trimeric Fn14 complexes are formed which recruit a single TRAF2-cIAP1/2 complex and reduces so the availability of the latter for NIK degradation. As a consequence, there is reduced NIK degradation leading to accumulation of NIK and eventually to signal-induced alternative NF-κB signaling. Since the Fn14-bound single TRAF2-cIAP1 and TRAF2-cIAP2 complexes remain largely inactive due to missing cIAP1/2 transactivation, there is no/poor classical NFκB signaling (middle panel). iii) In the presence of memTWEAK, ligated trimeric Fn14 complexes are formed, too but due to their high concentrations in the cell-to-cell contact zone these complexes cluster secondarily leading to the recruitment of several TRAF2-cIAP1/2 complexes to the Fn14 clusters. Again, this reduces the availability of TRAF2-cIAPs complexes for NIK degradation and triggers the alternative NF-κB pathway. However, since the Fn14-clusters bind several TRAF2-cIAP1/2 complexes, transactivation of cIAPs becomes possible leading eventually in classical NFκB signaling (right panel).

Sequestration of TRAF2-cIAP1/2 complexes and TRAF3 to the TWEAK-induced Fn14 signaling complex, as described above in context of the death receptor-Fn14 crosstalk, results therefore in reduced NIK degradation, NIK accumulation, p100 to p52 processing and eventually enhanced nuclear translocation of p52-containing NF-κB dimers (Wajant, 2013). Again, sTWEAK as well as membrane TWEAK efficiently trigger this signaling pathway (Roos et al., 2010). Fn14 also activates the classical NF-κB pathway by crucial help of TRAF2 and cIAPs (Figure 1). The trimeric TRAF2 molecule interacts with a single molecule of cIAP1 or cIAP2 (Mace et al., 2010; Zheng et al., 2010). Activation of cIAP1 and cIAP2 requires homotypic interaction/dimerization of their RING domain (Feltham et al., 2011). In cIAP1/2 monomers, however, the RING domain is intramolecularly inhibited by shielding by the BIR3, UBA and CARD domains (Dueber et al., 2011; Feltham et al., 2011; Lopez et al., 2011). The need of RING domain dimerization for cIAP1/2 activation suggests that TRAF2_3_-cIAP1 and TRAF2_3_-cIAP2 complexes have no or only a low E3 ligase activity. Accordingly, clustering of trimeric TWEAK-Fn14 complexes seems necessary to promote the proximity of two or more TRAF2_3_-cIAP1/2 complexes to favor RING domain dimerization and activation of the classical NF-κB pathway (Figure 1). Additionally, we observed that soluble TWEAK trimers which bind three Fn14 molecules largely fail to trigger the classical NF-κB pathway despite robust TRAF2 recruitment while oligomerized soluble TWEAK trimers robustly activate this response (Roos et al., 2010). The involvement of the IKK complex, TRAF2, cIAPs and the IKK-stimulating kinase TAK1 in the activation of the classical NF-κB pathway by Fn14 has been proven by using Fn14 knockout cells, siRNA experiments and pharmacological inhibition but is poorly understood in detail (Saitoh et al., 2003; Kumar et al., 2009; Varfolomeev et al., 2012). Intriguingly, the E3 ligase activity of the TRAF2-cIAP1/2 complexes in classical and alternative NF-κB signaling are qualitatively different, promoting K48 ubiquitination to instruct proteasomal degradation of ubiquitinated proteins *versus* K63 ubiquitination to create docking sides for downstream signaling molecules. How the decision is made for the K48 or K63 type of ubiquitination by TRAF2 and the cIAPs is still largely unknown.

Noteworthy, TRAF2 and the structurally related protein TRAF1 form heterotrimers which recruit cIAP1 and cIAP2 even better than TRAF2 trimers (Zheng et al., 2010) and act in many aspects similar to the TRAF2-cIAP1 and TRAF2-cIAP2 complexes. TRAF1 is poorly expressed in most cells and is instead induced by NF-κB transcription factors (Wajant, 2013). Moreover, TRAF1, in contrast to many other NF-κB-regulated proinflammatory factors, such as IL8 and IL6, is not only robustly induced *via* the classical NF-κB pathway but also *via* the alternative NF-κB pathway. Thus, TRAF1 in contrast to most other NF-κB-regulated factors is already induced by sTWEAK (Carmona Arana et al., 2014). Interestingly, we furthermore observed that TRAF1 antagonizes TRAF2 degradation and thus maintains TRAF2-dependent signaling events (Wicovsky et al., 2009). It is therefore tempting to speculate that TRAF1 gains relevance in the adjustment of Fn14 activities when cells are chronically exposed to TWEAK.

## 3 TWEAK and Fn14 in cancer biology

Based on the ability to sensitize cells for TNF-induced cell death, the TWEAK/Fn14 system has obviously the potential to suppress together with TNF tumor development. Indeed, Vredevoogd et al. (2019) found a TNF response signature in patients responding to an immune checkpoint blockade and identified TRAF2 as a major factor preventing tumor cell death-induction by CD8^+^ T-cell produced TNF in animal models. Moreover, they mimicked the TNF-sensitizing effect of TRAF2 knockout in their models by Fn14 stimulation (Vredevoogd et al., 2019). Increased activity of the TWEAK/Fn14 system has also been associated with better outcome (overall-/disease-free survival) in patients suffering on colorectal cancer and was furthermore correlated with TWEAK-induced inhibition of *in vitro* invasiveness of colon cancer cell lines (Lin et al., 2012).

Most cellular activities engaged by the Fn14/TWEAK system, however, have the potential to promote cancer development. This in particular accounts for its ability to stimulate cell migration. TWEAK/Fn14-promoted cell migration and/or invasiveness have been reported for various tumor cell lines *in vitro*, including glioblastoma cells, NSCLC, ovarian cancer cells, breast cancer cell lines and androgen-independent prostate cancer cells (Tran et al., 2003; Willis et al., 2008; Dai et al., 2009; Huang et al., 2011; Lin et al., 2012; Whitsett et al., 2012; Asrani et al., 2013; Whitsett et al., 2014; Cheng et al., 2015; Jandova et al., 2015; Armstrong et al., 2016; Wang et al., 2018; Hu et al., 2019; Li et al., 2019). Moreover, there is also evidence from *in vivo* models that the TWEAK/Fn14 system can act as a crucial driver of tumor cell migration and metastasis. A549 cells overexpressing Fn14 showed enhanced lung metastasis upon tail vein injection in mice (Whitsett et al., 2012) and H460 tumor cells over-expressing Fn14 showed more metastases in lung, liver and lymph nodes compared to mice challenged with H460 cells with low Fn14 levels (Jandova et al., 2015). Similarly, in a prostate cancer xenograft model with bone metastatic DU145 Ras^G37^ tumor cells, knockdown of Fn14 resulted in significantly reduced numbers of liver and brain tumors (Yin et al., 2014). The relevance of the TWEAK/Fn14 system for tumor cell migration and invasiveness is particular well understood in glioblastoma. Overexpression of Fn14 in the glioblastoma cell lines SF767 and T98G resulted in enhanced cell motility, membrane ruffling and lamellipodia formation (Tran et al., 2003). Moreover, TWEAK induces in glioblastoma cell lines sequential activation of the Rho GTPase Cdc42 and Rac1 along with Rac1-dependent cell migration (Tran et al., 2003; Fortin et al., 2012). In line with these findings, the guanine nucleotide exchange factors (GEFs) Ect2 and Trio, which promote the conversion of Cdc42 and Rac1 to their GTP-bound active state, have been crucially involved in TWEAK-induced cell migration in the T98G glioma cell line (Fortin et al., 2012). Similarly, it has been demonstrated that RhoA and the RhoA-specific GEF Src homology 3 domain-containing guanine nucleotide exchange factor (SGEF) mediate TWEAK-induced cell migration in the glioma cell lines U87 and U118 (Fortin Ensign et al., 2013). RhoA and SGEF but also the Cdc42/Ect2-Rac1/Trio axis furthermore mediate TWEAK-induced lamellipodia formation in T98G cells (Fortin et al., 2012; Fortin Ensign et al., 2013). In overexpression experiments co-immunoprecipitation of Fn14 and the aforementioned Rho-GTPases and GEFs have been observed (Fortin et al., 2012; Fortin Ensign et al., 2013) opening the possibility that these factors are part of the TWEAK-induced Fn14 signaling complex. In line with this idea, it has been found that TRAF2 and SGEF interact constitutively and recruited to Fn14 in a TWEAK-dependent manner. Furthermore, it has been described that TRAF2 knockdown inhibits TWEAK-induced cell migration in U87 and U118 cells (Fortin Ensign et al., 2013). Later studies revealed additionally that TWEAK not only generally stimulates cell motility in glioma cells but also chemotaxis. Accordingly, it has been reported that TWEAK induces activation of the non-receptor tyrosine kinase Lyn and that the knockdown of the latter abrogates TWEAK-induced chemotactic migration in T98A and A172 glioma cells as well as Rac1 activation (Dhruv et al., 2014). It is worth mentioning that SGEF expression is upregulated in glioma cell lines in response to Fn14 activation and is not only required for cell migration but also seems to contribute to resistance to DNA damaging chemotherapeutic drugs, such as temozolomide (TMZ), which is used to treat glioblastoma (Ensign et al., 2016) In fact, patient derived glioblastoma cells with acquired TMZ resistance display higher Fn14 expression and enhanced migratory activity compared to TMZ-sensitive cells (Hersh et al., 2018a; Hersh et al., 2018b). In accordance with a central role of the TWEAK/Fn14 system for the motility and aggressiveness of glioblastoma cells, Fn14 expression is strongly increased in gliosarcoma, a particularly aggressive form of high grade glioma, isocitrate dehydrogenase 1 (IDH1) wild-type glioblastoma, which have a poorer prognosis than IDH1 mutated glioblastoma, mesenchymal type glioblastomas and recurrent glioblastoma (Tran et al., 2006; Fortin et al., 2009; Perez et al., 2016; Hersh et al., 2018a). Moreover, experimentally generated proneural-like gliomas (PDGF-A expression, p53 knockdown) in rats convert to highly invasive brain cancer with constitutive classical and alternative NF-κB signaling upon introduction of Fn14 (Connolly et al., 2021).

Fn14 activities, however, also contribute to tumor development beyond improving cell migration. For example, Fn14 is induced by proangiogenic proteins such as fibroblast growth factor (FGF)-2 and vascular endothelial growth factor (VEGF)-A and *vice versa* TWEAK and Fn14 can promote the expression of FGF-2 and VEGF (Donohue et al., 2003; Dai et al., 2009). In line with this reciprocal regulation, it has been observed that TWEAK-expressing Hek293 cells elicit enhanced angiogenesis in nude mice (Ho et al., 2004). There is furthermore evidence from an orthotopic model of urothelial carcinoma that ALKBH3 in cooperation with an autocrine TWEAK-Fn14 loop maintains expression of Fn14 and VEGF in tumor vessels to promote angiogenesis and tumor progression (Shimada et al., 2012).

## 4 Biologicals targeting the TWEAK/Fn14 system

Considering the complex biology of Fn14 both the inhibition of TWEAK or Fn14 and the activation of Fn14 can have beneficial therapeutic effects (Aggarwal et al., 2012). The interference with Fn14 activity is straightforwardly possible by the help of anti-TWEAK or anti-Fn14 antibodies preventing TWEAK-Fn14 interaction or by soluble fusion proteins containing the extracellular domain of Fn14. The efficacy of TWEAK- and Fn14-blocking antibodies and a Fn14–Fc decoy receptor has been demonstrated in various preclinical disease models (Table 2).

**TABLE 2:** Examples of the use of TWEAK- and Fn14 antagonists in preclinical disease models.

Antagonist	Disease model	Effect	References
Fn14-Fc	SLE developing sanroque mice	Less germinal center formation	Min et al. (2016)
Fn14-Fc	ApoE −/− mice	Smaller, fewer and less fibrotic atherosclerotic plaques	Schapira et al. (2009)
Fn14-Fc	Middle cerebral artery occlusion in mice	Reduced infarct volume Reduced cerebral edema Improved motor activity	Yepes et al. (2005), Zhang et al. (2007)
Fn14-Fc	β-glucan–induced arthritis in SKG mice	Reduced disease score	Park et al. (2017)
Anti-TWEAK	Autosomal dominant polycystic kidney disease	Improved renal function Improved survival	Cordido et al. (2021)
Anti-TWEAK (P2D10)	ApoE −/− mice	Reduced atherosclerotic burden Delayed plaque progression	Fernández-Laso et al. (2017)
Anti-Fn14 18D1-dead	Acute graft-versus-host disease	Reduced intestinal cell death Improved survival	Chopra et al. (2015)
Anti-Fn14 18D1-dead	Intraportal injection xenotransplant model of HCT116 metastasis	Reduced metastasis Improved survival	Trebing et al. (2014)
Anti-Fn14 ITEM-2	Renal ischemia reperfusion injury	Reduced accumulation of neutrophils and macrophages Less tubular cell apoptosis. Prolonged survival	Hotta et al. (2011)
Anti-TWEAK (P5G9)	Chronic graft-versus-host disease-induced lupus	Reduced cytokine/chemokine production and reduced proteinuria	Zhao et al. (2007)
Anti-TWEAK (MTW-1)	Collagen-induced arthritis	Reduced disease score	Kamata et al. (2006)
Anti-TWEAK (P5G9)	Collagen-induced arthritis	Reduced disease score and less synovial angiogenesis	Perper et al. (2006)

**FIGURE 2:**
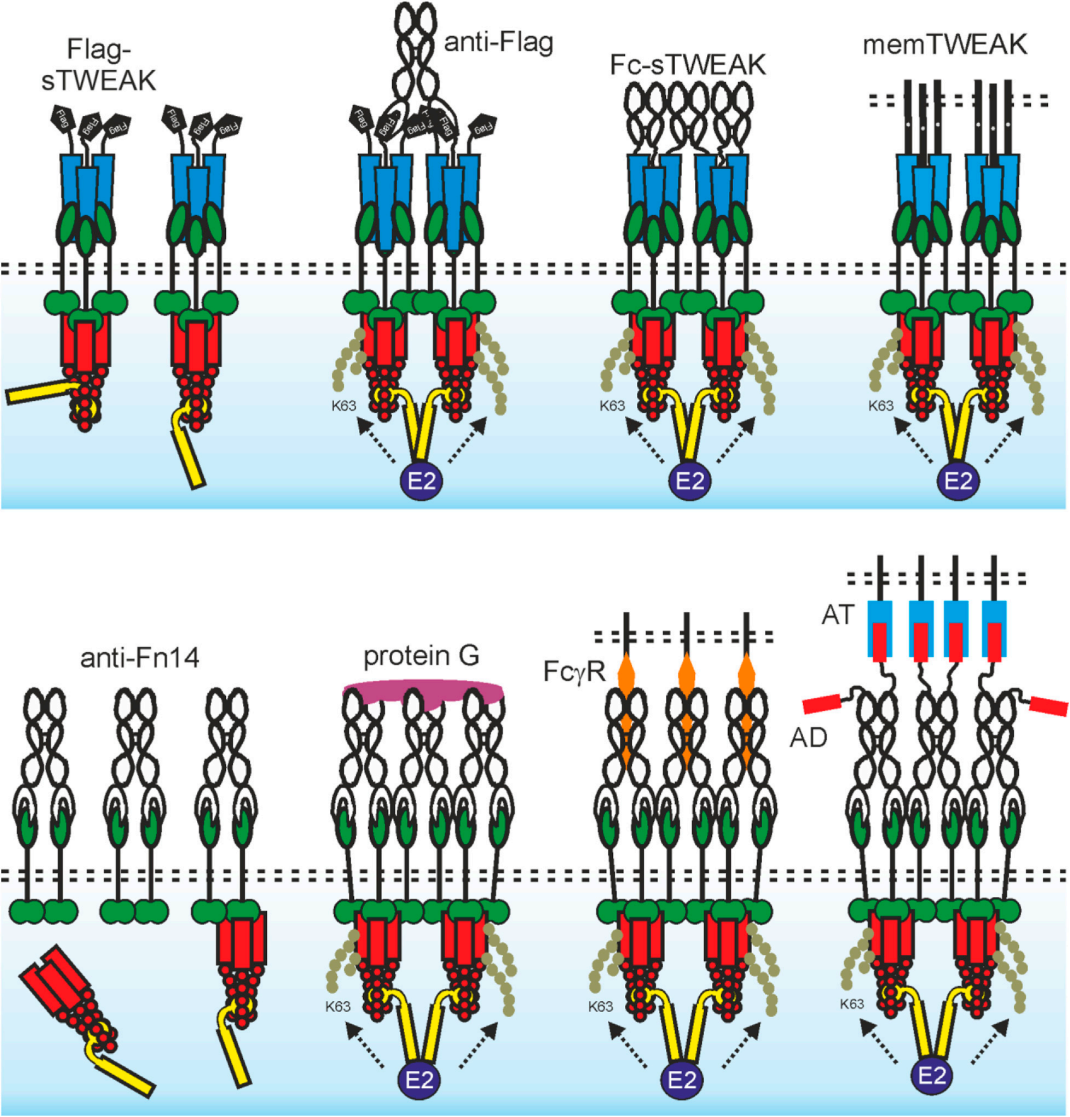
The agonistic quality of TWEAK trimers and anti-Fn14 antibodies is determined by oligomerization and membrane-association. Upper panel: An sTWEAK trimer recruits three Fn14 molecules. The Fn14 trimers assembled by sTWEAK in turn recruit complexes of a TRAF2 trimer with one cIAP1 or cIAP2 molecule. This results in reduced availability of these molecules for other binding partners but does not trigger efficient cIAP transactivation. If the proximity of two (or more) of these sTWEAK-associated Fn14 trimers is enforced, e.g., by use of antibody crosslinking (e.g., Flag-tagged sTWEAK with anti-Flag antibody) or by genetic fusion with an oligomerization domain (e.g., Fc domain), this results in close neighborhood of two (or more) TRAF2-cIAP1/2 complexes and thus in cIAP transactivation and classical NF-κB signaling (see also Figure 1). Due to the high concentrations of trimeric Fn14 complexes in the cell-to-cell contact zone with memTWEAK expressing cells, there is spontaneous clustering of the Fn14 complexes and again cIAP transactivation due to the tight neighborhood of TRAF2-cIAP1/2 complexes. Lower panel: An anti-Fn14 antibody recruits two Fn14 molecules. The anti-Fn14-bound Fn14 dimers recruit one TRAF2-cIAP1/2 complex but less efficient than an sTWEAK trimer. Thus, there is some reduction in the cytosolic availability of TRAF2-cIAPs and therefore alternative NF-κB signaling despite weaker as with sTWEAK (see upper panel and Figure 1). If the linkage of three (or more) of these anti-Fn14-associated Fn14 dimers is enforced, e.g. by protein G crosslinking this also induces neighborhood of several TRAF2-cIAP1/2 complexes and thus cIAP transactivation and classical NF-κB signaling. Due to the high concentrations of dimeric Fn14 complexes in the cell-to-cell contact zone with cells decorated with FcγR-bound anti-Fn14 antibodies (or anchoring target (AT)-bound anti-Fn14 antibody fusion proteins with an anchoring domain (AD), there is again spontaneous clustering of the Fn14 complexes and cIAP transactivation.

The usefulness of recombinant soluble TWEAK is limited in several ways. Soluble TWEAK trimers are rapidly cleared from the circulation in mice with a half-life of less than 1 h (Muller et al., 2010). Furthermore, as already discussed, it does not comprehensively stimulate Fn14. Both limitations of sTWEAK can be overcome by genetic fusion with heterologous protein domains. For example, the serum retention of sTWEAK can be strongly enhanced by genetic fusion with serum albumin (Muller et al., 2010). Connecting two or more sTWEAK trimers or enabling cell surface anchoring of sTWEAK furthermore confer membrane TWEAK-like activity (Figure 2). Thus, a Fc-TWEAK fusion protein forms hexamers, has memTWEAK-like activity and presumably act by enforcing close proximity of two liganded Fn14 trimers (Roos et al., 2010). The Fc domain furthermore greatly improves serum retention. It has been furthermore demonstrated that a cell surface antigen-recognizing trimeric scFv-sTWEAK fusion protein acts as sTWEAK in the absence of the cell surface antigen but convert to a molecule with memTWEAK activity when bound to the cell surface exposed target of its scFv domain (Roos et al., 2010). Recombinant TWEAK variants have been used in some preclinical models. However, expression of rec. TWEAK seems to be less efficient than that of antibodies and in general, there is less experience with the translational development and approval of recombinant TNFLs.

Anti-Fn14 antibodies alone have no or only limited agonism (Figure 2). However, anti-Fn14 antibodies regularly gain comprehensive and strong agonism upon cross-linking with secondary antibodies or protein G (Salzmann et al., 2013; Trebing et al., 2014). This resembles the situation of sTWEAK which gains maximal and comprehensive memTWEAK-like activity after oligomerization (see above). The idiotype of an anti-TNFR antibody and thus the recognized epitope is typically considered as the main factor determining agonistic activity. However, there is now broad evidence that antibodies specific for a subgroup of the TNFRSF, including Fn14, regularly gain high agonistic activity by FcγR-binding (Wajant, 2015) (Figure 2). Indeed, the non-competitive anti-Fn14 antibodies PDL192 and 5B6 and the blocking antibodies P4A8 and 18D1 recognize different epitopes within the extracellular domain of Fn14. All these antibodies show, however, strong classical NF-κB activation after FcγR-binding (Trebing et al., 2014). Moreover, all combinations of antibody isotype and FcγR type which show binding also result in significant Fn14 activation (Medler et al., 2019). The isotype is therefore obviously only of importance for agonistic activity of an anti-Fn14 antibody in so far that it determines the ability to bind FcγRs (Medler et al., 2019). Importantly, the maximal Fn14 activity achieved with FcγR-anchored antibodies is comparable to those elicited by memTWEAK (Medler et al., 2019). In sum, it can be asserted that oligomerization or FcγR-binding is regularly required, but also sufficient, for the agonism of antibodies targeting Fn14 and that it is largely the sheer cell surface anchoring that constitutes the agonism of FcγR-bound antibodies while FcγR-specific activities are largely irrelevant. With respect to the potential translational exploitation of anti-Fn14 antibodies as agonists, it is important to realize that the required FcγR binding comes along with several limitations. First, due to poor availability of FcγR-expressing cells and/or low cellular FcγR expression levels, an only suboptimal agonistic activity might be reached *in vivo*. Second, FcγR-mediated effects triggered by the antibody-FcγR interaction can counteract the anticipated therapeutic effects which are actually aspired by the anti-Fn14 antibody treatment. For example, the aim of triggering Fn14-mediated inflammatory activity in tumors might be antagonized by stimulation of the anti-inflammatory FcγRIIB or FcγRIIIA-mediated ADCC of non-transformed Fn14-expressing cells. Third, considerable antibody doses are typically required to overcome competition with serum IgGs for FcγR binding. Worth mentioning the FcγR-binding related limitations of anti-Fn14 antibodies can be overcome by modification in a way enabling them to anchor to cells in a FcγR-independent fashion. Thus, anti-Fn14 antibody fusion proteins having an anchoring domain (e.g., a scFv domain) enabling binding to a cell surface-exposed anchoring target (AT) distinct from FcγRs display strong Fn14 stimulation when presented bound to the AT but not in presence of anchoring target-negative cells (Figure 2). Anchoring dependent Fn14 agonism was also achieved with FcγR-binding defective antibody mutants and Fab_2_-fragments (Medler et al., 2019; WO2019129644 A1). Worth mentioning, anti-Fn14 immobilized on gold nanoparticles are also highly agonistic (Aido et al., 2021). In this case, it is, however, unclear whether the agonism is due to oligomerization of several anti-Fn14 antibody molecules on a single particle or the surface-associated mode of presentation.

## 5 Targeting of the Fn14/TWEAK system for the treatment of cancer

The high tumor-associated expression of Fn14 (Table 1) along with its proliferation-, cell migration- and angiogenesis promoting activities lead to the consideration of the TWEAK/Fn14 system as a promising target for tumor therapy. In this context, two major approaches have been discussed and evaluated. First, the exploitation of Fn14 expression to direct anti-cancer effector activities to the tumor and to destroy Fn14-expressing tumor cells. Second, blockade of Fn14 or TWEAK to inhibit protumoral activities of these molecules. A third, yet not or only poorly investigated possibility is to activate Fn14 to exploit its proinflammatory and cell death sensitizing properties to destroy tumor cells and/or the tumor microenvironment.

### 5.1 Exploitation of Fn14 as tumor-associated antigen to trigger immune effector functions and to guide antitumoral payloads

The principle usefulness of targeting Fn14 as a tumor-associated tumor antigen has been successfully demonstrated in preclinical studies with antibodies, antibody drug conjugates and CAR T-cells specific for Fn14 (Table 3).

**TABLE 3:** Anti-tumor activity of Fn14-targeting biologicals.

Biological	Mode of action/payload	Model/treatment	Effect	References
Anti-Fn14 BIIB036	ADCC and/or FcγR-dependent agonism	Xenograft (WiDr, MDA-MB-231, NCI-N87, Hop-62)Athymic mice, patient derived primary tumors	Tumor growth inhibition	Michaelson et al. (2011), Michaelson et al. (2012)
Anti-Fn14 Enavatuzumab	ADCC and/or FcγR-dependent agonism	Xenograft (A253, MiaPaCa2, A375, MCF7, HCC70, SN12C)	Tumor growth inhibition	Chao et al. (2013), Culp et al. (2010), Ye et al. (2017)
Anti-Fn14 18D1-enhanced	ADCC	Renca	Reduced metastasis	Trebing et al. (2014)
Anti-Fn14 18D1-dead	Fn14 blockade	HCT116	Reduced metastasis	Trebing et al. (2014)
ADC ITEM4-rGel	Gelonin-linked	Xenograft (T-24)	Tumor growth inhibition	Zhou et al. (2011)
ADC ITEM4-rGel SGZ (dimerized scFv:IT4-rGel)	Gelonin-linked	Xenograft (MDA-MB-435)	Tumor growth inhibition	Zhou et al. (2013a)
SGZ	Gelonin-linked	Xenograft (MDA-MB-231-Luc)	Tumor growth inhibition	Zhou et al. (2013b)
GrB-TWEAK	Granzyme-B linked	Xenograft (HT29, MDA-MB-231)	Tumor growth inhibition	Zhou et al. (2014).
GrB-scFv:IT4	Granzyme-B linked	Xenograft (HT29, MDA-MB-231)	Tumor growth inhibition	Zhou et al. (2014).
Paclitaxel anti-Fn14 ITEM-4 nanoparticles	Paclitaxel	Xenograft (231-Luc)	Tumor growth inhibition	Dancy et al. (2020)
Anti-Fn14 BAY-356	Kinesin spindle protein inhibitor	Xenograft (NCI-H292, BFX1218)	Strongly reduced tumor growth	Lerchen et al. (2018)

The anti-Fn14 antibody BIIB036 (humanized version of anti-Fn14 P4A8) showed a strong anti-tumor effect on various xenografted tumor cell lines (Table 3) and patient derived primary tumors (Michaelson et al., 2011; Michaelson et al., 2012). *In vitro* studies proved efficient ADCC induction by BIIB036 (Michaelson et al., 2011). Similarly, the anti-Fn14 antibody enavatuzumab (humanized version of anti-Fn14 PDL192) displayed anti-tumor efficacy on several xenografted tumor cell lines and again this correlated in most cases with the ability of the antibody to interact with FcγRs and ADCC induction (Culp et al., 2010; Chao et al., 2013; Ye et al., 2017). The anti-Fn14 antibody 18D1 furthermore showed a FcγR-dependent antitumoral effect in the syngeneic Renca lung metastasis model (Trebing et al., 2014). As discussed already above, anti-Fn14 antibodies regularly acquire high memTWEAK-mimicking agonistic activity upon FcγR-binding. The FcγR-dependent antitumoral effects of BIIB036, enavatuzumab and 18D1 are thus not necessarily indicative for an exclusive and obligate relevance of FcγR effector mechanisms but could, at least in some cases, also reflect the stimulation of Fn14 signaling. In fact, enavatuzumab inhibited xenotransplanted BT549 tumor cells and this correlated with the ability of these tumor cells to respond to enavatuzumab with activation of the classical and alternative NF-κB pathway (Purcell et al., 2014). In sum, Fc-FcγR-interaction dependent antitumor activity of anti-Fn14 antibodies has been demonstrated in various models *in vivo*, however, it has, not been clarified yet to which extend this antitumor activity is due to stimulation of FcγR-induced effector functions, e.g., ADCC, and/or the enhanced agonism of FcγR-bound anti-Fn14 antibodies and Fn14-mediated signaling. In fact, the two mechanisms not necessarily act mutually exclusive. As discussed before, Fn14 can also elicit protumoral activities. Such activities could be stimulated by FcγR-interacting anti-Fn14 antibodies, too, but should be of transient nature in the case of antibodies triggering destruction of the Fn14-expressing cells. The enthusiasm about the potential of Fn14-targeting with FcγR-interacting antibodies, however, is dampened through a phase I clinical study with enavatuzumab in patients with solid malignancies revealing dose-limiting liver and pancreatic toxicities (Lam et al., 2018).

Immunotoxins composed of geolin chemically linked to the anti-Fn14 antibody ITEM-4 or genetically fused to an ITEM-4 derived scFv were also found to show strong antitumor effects to xenotransplanted cancer cells (T-24, MDA-MB4-432 melanoma cells, MDA-MB-231 breast cancer cells) which were otherwise poorly sensitive for ITEM-4 (Zhou et al., 2011; Zhou et al., 2013a; Zhou et al., 2013b). Likewise, a Fn14-specific antibody-drug conjugate with an aglycosylated variant of the anti-Fn14 antibody Bay-365 and a kinesin spindle protein inhibitor showed antitumor efficacy against xenotransplanted NCI-H292 cells (Lerchen et al., 2018). Granzyme B fusion proteins of sTWEAK or an anti-Fn14 scFv domain showed selective cytotoxicity on Fn14-expressing cells and inhibited tumor growth of xenografted HT29 and MDA-MB-231 tumor cells, too (Zhou et al., 2014). The usefulness of Fn14 as a target to direct cytotoxic drugs to tumors has also been reported with ITEM-4 conjugated paclitaxel-loaded nanoparticles which outperformed nontargeted nanoparticles in preclinical models of primary and brain metastatic triple-negative breast cancer (Dancy et al., 2020). In view of the fact that anti-Fn14 antibodies immobilized on gold nanoparticles acquire agonistic activity (Aido et al., 2021), it appears possible that engagement of Fn14 signaling contributed to the superior antitumoral activity of Fn14-targeted paclitaxel-loaded nanoparticles.

### 5.2 Inhibition of tumor promoting activities of the TWEAK/Fn14 system

Considering the protumoral effects of Fn14, especially its cell migration-stimulating activity (see 3.), there is also evidence for the idea that blockade of the Fn14/TWEAK system can elicit antitumoral effects. It has been observed in an intraportal injection xenotransplant model of hepatic metastasis with HCT116 colon cancer cells that a FcγR-binding defective variant of the blocking anti-Fn14 antibody 18D1 reduces metastasis (Trebing et al., 2014). Regression of tumor development has also been reported for the TWEAK-neutralizing antibody RG7212 in several xenograft tumor models (ACHN, MDA-MB-231, Caki-1, Calu-3) with high Fn14 expression (Yin et al., 2014). Moreover, RG7212 showed very good tolerability and evidence for tumor regression in heavily pretreated patients with solid tumors in a phase I monotherapy study (Lassen et al., 2015). Since there is yet no reliable possibility to distinguish between endogenous sTWEAK- and memTWEAK-induced effects, it is fully unclear whether the beneficial effects of the inhibition of the TWEAK-Fn14 interaction in these studies reflect protumoral activities of soluble or membrane TWEAK or of both forms of the ligand. It is also unclear whether the antitumoral effects of TWEAK/Fn14 blockade in the aforementioned models mirror general protumoral activities of Fn14 or whether the net effect of the Fn14 activation on tumor development changes if a certain threshold of Fn14 activity is exceeded. Indeed, there is evidence for different thresholds of Fn14 stimulation for enhancement of TNFR1-induced cell death and activation of the alternative NF-κB pathway (Salzmann et al., 2013). In the absence of FcγR-binding, anti-Fn14 antibodies, similar to sTWEAK, significantly trigger p100 processing, the hallmark of the alternative NF-κB pathway. However, while sTWEAK is also sufficient to sensitize for TNF receptor 1-induced cell death, free anti-Fn14 antibodies fail to do so or even block the corresponding sTWEAK response (Salzmann et al., 2013). Therefore, it appears that oligomerized or FcγR-bound Fn14 antibodies comprehensively mimic memTWEAK, while free bivalent anti-Fn14 antibodies only resemble sTWEAK in a response selective manner. One have to consider that *in vivo* sTWEAK is presumably dominant over memTWEAK due to the efficient processing of memTWEAK to sTWEAK by furin proteases. Therefore, even in cases where the endogenous activity of the TWEAK/Fn14 system elicits protumoral effects, strong exogenous Fn14 stimulation, especially with memTWEAK mimicking Fn14 agonists, might nevertheless display antitumoral activities.

### 5.3 Antitumoral potential of exogenous Fn14 activation

Since FcγR-binding regularly converts anti-Fn14 antibodies into strong membrane TWEAK-mimicking agonists one could mean that the potential of triggering antitumoral effector functions should be evident from the various studies with anti-Fn14 antibodies described above. Indeed, there is initial evidence from *in vivo* studies with enavatuzumab that anti-Fn14-induced “agonistic” chemokine production result in enhanced migration of myeloid cells into the tumor microenvironment where then the same anti-Fn14 antibody acts antitunoral by its effector activity (Ye et al., 2017). However, the full antitumoral potential of anti-Fn14 agonism is presumably underestimated from studies with anti-Fn14 antibodies. Besides the fact that antibody-linked effector activities might mask antitumoral activities of Fn14 molecules stimulated by FcγR-bound anti-Fn14 antibodies, one have to consider that most anti-Fn14 antibodies have been primarily studied in xenograft models (Table 3) without a functional adaptive immune system. It is thus tempting to speculate that antitumor activities related to proinflammatory Fn14 signaling engaged by FcγR-bound anti-Fn14 antibodies have been underestimated, yet. The antitumoral activity of pure Fn14 agonists, however, has been poorly addressed yet *in vivo*. This is not at least due to the fact that there are yet no agonistic Fn14 antibodies displaying FcγR-independent agonism. More elaborated studies with recombinant TWEAK variants are therefore necessary in the future to evaluate the therapeutic potential of agonistic Fn14 targeting. A first promising study in this respect revealed strong inhibition of tumor development from MDA-MB-231 tumor cells injected in nude mice after treatment with Fc-TWEAK or adenoviral administration of TWEAK (Michaelson et al., 2011). In view of very recent results, which identified TRAF2 and the cIAPs as major targets facilitating CD8^+^ T-cell elimination of tumor cells in a TNF-dependent manner in patients responding to immune checkpoint blockade (Vredevoogd et al., 2019), it appears furthermore promising to evaluate the potential of Fn14 agonists in combination therapies with checkpoint inhibitors.

## 6 Targeting of the Fn14/TWEAK system for the treatment of cancer- and cancer treatment-associated complications

As already briefly discussed, Fn14-related activities crucially contribute to complications arising from overshooting and/or chronic repair processes. In view of the concept of cancer as a wound that does not heal, it appears possible to target the TWEAK/Fn14 system in cancer not with the aim to attack the tumor development process itself but rather to attenuate detrimental effects of cancer development and cancer treatments on healthy parts of the body. Indeed, there is evidence from animal models that Fn14 blockade can reduce tumor-associated cachexia or therapy-induced diarrhea (Johnston et al., 2015; Sezaki et al., 2017). Furthermore, in good accordance with its sensitizing effect for cytotoxic TNFR1 signaling and the well established high sensitivity of the intestinal epithelium for TNF, it was found that a blocking anti-Fn14 antibody has a strong protective effect on graft-versus host disease in the intestine (Chopra et al., 2015). Notably, the GvHD protective effect of Fn14 blockade allowed efficient treatment of syngeneic tumors by adoptive transfer of allogeneic T-cells (Chopra et al., 2015).
